# Microbial pesticides – challenges and future perspectives for testing and safety assessment with respect to human health

**DOI:** 10.1186/s12940-024-01090-2

**Published:** 2024-05-29

**Authors:** K. Wend, L. Zorrilla, F. M. Freimoser, A. Gallet

**Affiliations:** 1 German Federal Institute for Risk Assessment, Max-Dohrn-Str. 8-10, Berlin, 10589 Germany; 2 Bayer Crop Science, 700 Chesterfield Parkway West, Chesterfield, MO 63017 USA; 3 Agroscope, Research Division Plant Protection, Route de Duillier 60, Nyon 1, 1260 Switzerland; 4 Université Côte d’Azur, CNRS, INRAE, ISA, Sophia-Antipolis, 06903 France

**Keywords:** Microorganism, Microbial pesticides, Human health, Pesticide regulation, New approach methods

## Abstract

Plant protection measures are necessary to prevent pests and diseases from attacking and destroying crop plants and to meet consumer demands for agricultural produce. In the last decades the use of chemical pesticides has largely increased. Farmers are looking for alternatives. Biopesticides should be considered a sustainable solution. They may be less toxic than chemical pesticides, be very specific to the target pest, decompose quickly, and be less likely to cause resistance. On the other hand, lower efficacy and higher costs are two disadvantages of many biopesticides. Biopesticides include macroorganisms, natural compounds and microorganisms. Microbial pesticides are the most widely used and studied class of biopesticides. The greatest difference between microbial and chemical pesticides is the ability of the former to potentially multiply in the environment and on the crop plant after application. The data requirements for the European Union and the United States Environmental Protection Agency are highlighted, as these regulatory processes are the most followed in regions where local regulations for biopesticide products are not available or vague. New Approach Methods already proposed or harmonized for chemical pesticides are presented and discussed with respect to their use in evaluating microbial pesticide formulations. Evaluating the microbials themselves is not as simple as using the same validated New Approach Methods as for synthetic pesticides. Therefore, the authors suggest considering New Approach Method strategies specifically for microbials and global harmonization with acceptability with the advancements of such approaches. Further discussion is needed and greatly appreciated by the experts.

## Introduction

### Microbial pesticides in plant protection

Plant protection measures are necessary to prevent pests and diseases from attacking and destroying crop plants and to meet consumer demands for agricultural produce. In the last decades the use of chemical pesticides has largely increased [1]. Farmers are looking for alternatives due to regulations reflecting the “Green Deal” and emerging resistance issues [2, 3]; alternative solutions are being considered and sought after. Biopesticides should be considered as one of several sustainable solutions in a grower’s toolbox. They may be less toxic than chemical pesticides, might be very specific to the target pest, decompose quickly and may be less likely to develop resistance [4]. On the other hand, a lower efficacy and higher costs are two main disadvantages of many biopesticides, indeed their efficacy can drop down to 50% compared to the 80% estimated for chemical pesticides [5, 6].

For future applications, combinations of chemical pesticides and biopesticides may thus be able to combine the best of both approaches and allow reducing the overall amount of synthetic chemical pesticides that are applied in the environment.

Biopesticides may include macroorganisms (such as parasitoids and predators), natural compounds (such as plant extracts) and microorganisms (such as fungi, bacteria, viruses, protozoans). Microbial pesticides are the most widely used and studied class of biopesticides, more sustainable than chemical-based pesticides with similar risk to chemical-based pesticides related to their authorized labelled use [7, 8]. Microbial pesticides antagonise plant pathogens and pests by different mechanisms that include the production of toxins, secretion of enzymes, volatile compounds, direct colonization or consumption of the host, induction of resistance in crop plants, and the competition for nutrients and space [9]. Microbial antibiotics, antifungals or toxins produced by microorganisms can display many interesting properties: they can be produced in situ, be highly specific to a given target, often act at relatively low concentrations, or are biodegradable (or a combination of all these characteristics). However, beside these properties, there can be unintended effects on non-target organisms. The limited shelf life of many biocontrol agents due to their limited viability or products being conserved in liquid or dry formulations is a disadvantage. A shelf life of at least 18 months is considered a standard and a key aspect for a successful biopesticide product [10–13]. The greatest difference between microbial and chemical pesticides is the ability of the former to potentially multiply in the environment and on the crop plant after application. This may be considered as Janus-faced: on the one hand, the persistence of a microorganism in the environment can result in prolonged pest or disease control (e.g., an inoculative release strategy uses small numbers of microorganisms that are periodically applied, and will reproduce and establish a permanent population for longer-term control). On the other hand, if microorganisms may encounter favourable conditions, this can foster their proliferation and the production of potentially toxic metabolites in the environment. However, the species used in most microbial biopesticides are already present in the environment [14]. Possible potential adverse effects must be assessed and a testing strategy to confirm the safety for human and environmental health is required.

An assessment of the global market sales of agricultural chemicals and biopesticides indicates a growing potential for biopesticides. In 2023 the market sale for pesticides and other agricultural chemicals was USD 98.42 billion with an expected sale in 2027 of USD 139.42 billion and a resulting compound annual growth rate (CAGR) of 9.1% [15]. The global biopesticides market sales were much lower (2023: USD 6.7 billion; 2028: USD 13.9 billion, [16]). However, the CAGR of 15.9% indicates the potential of these types of plant pest and disease control products [16]. Considering over 55% of the globally marketed biopesticides are microbial pesticides [17], the high value of microbial pesticides is clear, and underlines the need to outline the challenges and future perspectives of assessing the potential risk of microbial pesticides.

The European Commission has recently provided the “Green Deal” in terms of reducing the use of chemical pesticides by 50% until 2030 [18]. Microbial biopesticides could contribute to achieving this goal due to their long-term growth over the past decade in contrast to chemical pesticides [19], but currently only account for less than 10% of the global pesticide market [20]. In Europe and as summarized by Helepciuc and Todor [5], the adoption of microbial pest control products is still slow. As the main cause, the lengthy, expensive, and two-stage procedure for the approval of biopesticides was identified. However, the authors also showed that the European Union (EU) had caught up with the United States (US) regarding research output, approved Microbial Pest Control Agents (MPCAs) and their regulatory approval procedures [5]. Further aspects that may influence the pesticide selection are economic motivation, education on the use of biopesticides to growers and the lack of training in pesticide management [21].

This manuscript is intended to display the current data requirements for microorganisms (MO) and products containing microorganisms as active substances. Improvements related to the data requirements are proposed and recommendations are presented for future strategies in consideration of non-animal testing. The need for microbial-specific guidelines will be especially highlighted. While there are well-defined Organization for Economic Cooperation and Development (OECD) testing guidelines for chemicals, few guidelines for microbial pesticides currently exist, and not all are fit-for purpose. Identifying appropriate data requirements is helpful to simplify the target-oriented development of guidelines and guidance documents for biopesticides.

### Examples of microbial pesticides

#### Bacteria

##### General overview

A range of bacterial species are globally registered and used as biocontrol agents against a variety of plant pests and diseases. Various spore forming *Bacillus* species (e.g., *B. amyloliquefaciens*, *B. subtilis and B. thuringiensis*) and *Streptomyces* as well as non-spore forming species in genera such as *Pasteuria* and *Pseudomonas* are used against soilborne plant pathogens, insect pests or plant parasitic nematodes [22–28]. Products based on *Bacillus* species consist of lyophilized spores mixed with additives and adjuvants.

Spores survive in stressful conditions and therefore are suitable for long-lasting storage and transport. Non-spore-forming bacteria are characterized by their ability to become dehydrated as live bacteria or concentrated in suspension. They can be stored at 4°C, or even at -25°C for conservation over several weeks. Both types of products have to be dissolved in water by the users before utilization.

The mechanisms of action of these bacteria are mainly achieved through the production of toxins or antibiotics by their secondary metabolites either stimulating host plant defense or killing the pathogens or the pests. In addition, some bacterial strains are thought to act by competing with the environmental niche of the pathogens. While toxins or antibiotics display a more specific mode of action, the stimulation of plant defense or the colonization of the root environment for instance has a broader range of action. Nevertheless, the exact mode of action is not always known and further studies are required to identify all mechanisms involved [6, 8, 29, 30].

The most widely used bacterium in agriculture is *Bacillus thuringiensis* and will be discussed in the following chapter.

##### Bacillus thuringiensis

Products derived from *Bacillus thuringiensis* are the second most sold insecticides (including chemical insecticides) worldwide with 32,000 tons sold in 2015 [31]. In 2019, *B. thuringiensis* products were authorized in 24 out of the then 28 EU Member States [5]. *B. thuringiensis* dominates the biopesticide market likely due to the specificity and limited acute impacts on beneficial and non-target organisms (receptor-mediated selectivity of *B. thuringiensis* Cry toxins) and lack of environmental persistence of Cry proteins [32–35].

*B. thuringiensis* was first identified in 1901 in Japan from a silkworm (*Bombyx mori*) and ten years later in Germany, from a population of flour moths (*Ephestia kuhniella*). The bacterium was quickly characterized for its specific entomopathogenic properties due to the presence of Cry toxins produced and embedded in parasporal crystal bodies during the sporulation of the bacteria [36]. Cry toxins are encoded by large plasmids only harbored by *B. thuringiensis* strains (and absent from the other bacteria of the *Bacillus cereus* group to which *B. thuringiensis* belongs) [37]. *B. thuringiensis* based insecticides were first commercialized as early as in the 1950s in the US [38]. The number of new *B. thuringiensis* subspecies discovered is still growing and a huge number of Cry toxins have been characterized [32, 34, 39–42] that display a broad toxicity towards many organisms like insects, worms, and gastropods [43, 44]. Each subspecies of *B. thuringiensis* produces between one and six different Cry toxins. The cocktail of Cry toxins produced by a given subspecies confers a specificity of action towards different phylogenetic orders [43]. However, only four wild type subspecies have been selected as commercial bioinsecticides due to their high specificity against crop pests: *B. thuringiensis* subsp*. kurstaki* and *B. thuringiensis* subsp. *aizawai* killing lepidopteran larvae, *B. thuringiensis* subsp. *morrisoni* (also named *tenebrionis*) killing coleopterans, and *B. thuringiensis* subsp. *israelensis* acting against mosquito larvae and targets dipterans, including nuisance and biting flies that emerge from aquatic habitats. Hence, most of the *B. thuringiensis* subspecies identified [41], although promising, are not currently used as biopesticides in part due to a lack of methods for assessment of their safety. In addition, many labs around the world are still screening for new *B. thuringiensis* strains having specific insecticidal activity (e.g., [45–47]), and these new strains will also need a well-defined evaluation framework for their safety assessment.

*B. thuringiensis* containing products are made of spores, toxin crystals and additives. Upon ingestion of *B. thuringiensis* bioinsecticides by the respective target species, Cry toxins are enzymatically activated in the insect midgut and subsequently form pores in the gut epithelium leading to osmotic lysis at alkaline pH. Additionally, the spores germinate and, due to the Cry toxin-induced holes in the gut epithelium, bacterial cells invade the internal body cavity, inducing septicemia and the targeted insect larva dies within 2 to 3 days [48, 49]. Noteworthy, the Cry toxin toxicity relies mainly on the presence of specific receptors capable to bind them, each family of Cry toxins requiring specific receptors [50]. Many studies have demonstrated that the gastrointestinal tract epithelial surface of non-target insects and mammals, including humans, lack specific high-affinity Cry protein receptors [39].

As the cry toxins are embedded in parasporal crystal bodies *B. thuringiensis* biopesticide formulations consequently contain *B. thuringiensis* spores (viable or non-viable). The presence of *B. thuringiensis* spores is a trigger of discussions about possible human health risks emanating from *B. thuringiensis* biopesticides. *B. thuringiensis* is part of the *B. cereus* group and displays similar genetic prerequisites as other members of this group, which are able to cause a foodborne toxico-infection associated with diarrheal symptoms [51–53], thus illustrating the importance of evaluating the genome as a first step to confirm the lack of the genes responsible for such illnesses in each microbial strain. This is true for any microbial species to be used in agricultural applications.

#### Fungi

Fungi were the first microorganisms to be envisioned as biopesticides. As early as the 1880’s, entomopathogenic fungi were studied and used in the field [54, 55]. Nowadays, the most commonly used entomopathogenic fungi are *Beauveria bassiana* and *Metarhizium robertsii* (formerly named *M. anisopliae*), as well as species of the genera *Hirsutella*, *Isaria*, *Lecanicillium*, *Paecilomyces* and *Verticillium* [56, 57]. More than 100 products containing specific strains of these fungi are registered as plant protection agents against all major groups of pest insects [58]. Fungi are not only used against insects, but also as biofungicides, bionematicides, or bioherbicides. Important biocontrol fungi comprise for example *Aureobasidium pullulans*, *Candida oleophila, Coniothyrium minitans, Duddingtonia flagrans, Gliocladium* and *Clonostachys* strains*, Myrothecium verrucaria,* as well as different species of the genus *Trichoderma,* and *Ulocladium oudemansii* [9, 59–66]. These organisms are usually used as preventive treatments in the field or in greenhouses and are applied to soil, the seeds, or sprayed in the phyllosphere. Besides entomopathogens, fungal-based biofungicides are the most important group of biocontrol fungi. The target phytopathogenic fungi and oomyctes include the causative agents of the most common and widespread plant diseases such as *Alternaria*, *Botrytis*, *Fusarium*, *Monilinia*, *Pythium*, *Phytophthora*, *Rhizoctonia*, and *Sclerotinia*.

Depending on the conditions and mode of action of these fungi, they exhibit different degrees of specificity. Opportunistic, generalist strains and specific antagonists are both used for biocontrol applications and have different advantages and disadvantages for such applications [67]. Generalist and specialist strains may even be found within the same genus, as for example in *Metarhizium* [68]. In some cases, the degree of specificity correlates with nutritional requirements (i.e., species with complex nutritional requirements tend to have narrow host ranges), but this is not always the case [69]. Overall, and as compared to biocontrol fungi used against fungal plant pathogens, entomopathogens tend to exhibit some degree of host specificity. In many cases, specificity is rather determined by the competitiveness in a particular ecological niche or the timing of an interaction than a targeted effect against another organism [70, 71]. Specificity is a criterion when assessing a microbial biocontrol agent, but it also has to be assessed in the context of the intended application. The formulation or location and timing of applications can also make a product specific and reduce unwanted effects on non-target organisms.

Biocontrol mechanisms are relevant for the safety assessment of a microbial pesticide. An important concern in the evaluation process are toxins that may have deleterious effects on consumers [72]. Fungi use a variety of different mechanisms to antagonize plant pathogens, diseases, and pests. Overall, competition for nutrients and space, including biofilm formation, are the most important modes of action of biocontrol fungi which target plant pathogens [9, 73, 74]. Many fungal biocontrol agents also secrete hydrolytic enzymes, produce inhibitory volatiles or secrete toxins. In particular, insect pathogens, but also some fungi (including yeasts) that are used against pathogenic fungi, directly parasitize or colonize their hosts. One indirect mode of action is the induction of systemic resistance in host plants. Unlike other microorganisms and their toxins (see “Bacteria” and “Viruses” sections), biocontrol-used fungi are often sensitive to ultraviolet light which impede spore germination, decreasing their field efficiency and dampening their prolonged action [75, 76].

#### Viruses

Viruses of the baculoviridae family are mainly used as bioinsecticides because of their efficiency and of their high specificity towards particular insects such as Lepidoptera, Hymenoptera and Coleoptera, though their utilization in agriculture is mainly exclusively targeted toward lepidopteran larvae [5, 77]. The baculovirus genome is composed of a double-stranded circular DNA sizing from 80 to 180 kb. The genome is enclosed in a rod shape capsid made of a proteinaceous crystalline matrix protecting it from harmful environment. This last property facilitates the commercial use of baculoviruses because of the possibility of long-term storage at ambient temperature (until 18 months) [19].

Nevertheless, many caveats limit the expansion of the use of baculovirus-based insecticides. The main caveat so far is the high cost of production, which is based on the use of in vivo systems such as live pest larvae. Moreover, baculoviruses have a relatively low persistence after spraying due to ultraviolet light degradation, necessitating frequent applications [19]. Finally, the negative effect related to the high specificity is the appearance of resistance in the target insect populations, requiring the identification of new baculovirus strains.

Slightly more than 90 different genomes of baculoviruses have been sequenced so far [78], and this number will continue to increase in the next years. It is assumed that the different strains of baculovirus can infect around 700 different insect species, most of them being Lepidoptera. Baculovirus efficiency ranges from 80 to 98% of killed insects within 5 days. They usually infect their host through ingestion by first infecting and replicating within enterocytes which are released to the rest of the body where they can further infect many cell types. The high host specificity of baculoviruses makes them avirulent for operators, workers, consumers and non-target organisms.

Pests sensitive to baculoviruses are found in a variety of crops, such as soybean, cotton, maize, sorghum, chickpea, black tea, Paraguay tea and many more. Cydia pomonella granulovirus (CpGV) was one of the first baculoviruses identified and commercialized to fight codling moth, a strong apple pest, in the middle of the 1980’s. Among baculoviruses, Nucleopolyhedroviruses (NPV) are also broadly used to fight lepidopteran pests with strains specific to a given lepidopteran species. For example, HearNPV is used to kill *Helicoverpa armigera*, SpltNPV to kill *Spodoptera litura* or AucaMNPV to fight *Autographa californica* [19, 77, 79, 80].

## General information on microbial pesticide regulation and data requirements

### The microbial pesticide regulation process

Within this chapter the microbial pesticide regulation processes in the EU and in the US are described, as these regulatory processes are the most followed in regions where local regulatory regulations for biopesticide products are not available or are vague.

#### The microbial pesticide regulation process in the European Union

In the EU, harmonization of pesticide registration schemes was sought under EU directive 91/414/EC [81]. Within this directive, Part A of the data requirements focuses on chemical pesticides, while Part B describes data requirements including toxicological studies for microorganisms and viruses. This directive was subsequently repealed by EU Commission Regulation (EC) No 1107/2009 (getting into force in 2011) [82], along with corresponding data requirements (Commission Regulation (EU) No 544/2011 [83] and 545/2011 [84]). The data requirements from 2011 were updated in 2013 and 2022 (Commission Regulation (EU) No 283/2013 [85, 86] and Commission Regulation (EU) No 284/2013 [87, 88] for active substances and products, respectively. They follow a similar format as in the earlier directive; Part A defines chemical requirements and Part B is focused on microbial active substances and plant protection products containing them. Part B data requirements have been revised by EU Commission and the amendments entered into applicability in November 2022 (Commission Regulation (EU) No 2022/1439 and 2022/1440) with a transition period until May 2023 for active substances and November 2023 for products, where previous data requirements are still valid. The European commission further published Explanatory Notes for the implementation of the data requirements on microorganisms and plant protection products containing them in the framework of Regulation (EC) No 1107/2009 [89].

Following current regulations, microbial active substances are assessed by Member States of the EU. A lead Member State (termed Rapporteur Member State (RMS)) conducts the initial evaluation of applicant´s dossier with a possible review/support from another Member State (co-RMS). The revised dossiers are then circulated for further commenting by the other Member States and European Food Safety Authority (EFSA) and, if necessary, a peer review meeting is organized to discuss unresolved issues. An EFSA conclusion (e.g., *Beauveria bassiana* strain 203 EFSA conclusion 2020, [90]) and subsequently a Commission review report is created. The Commission’s Review Report reflects its proposal. Only after the microorganism is approved on EU level, applications for products containing microorganisms are considered for authorization at Member State level.

#### The microbial pesticide regulation process in the United States

The United States Environmental Protection Agency (US EPA), the agency that regulates biopesticides within the US, initially required data for biopesticides on a case-by-case basis, recognizing that the variety of biopesticides that could be registered called for more flexibility in data requirements and testing to determine safety. The US EPA has their own biopesticides division, Biopesticides and Pollution Prevention Division (BPPD), which is responsible for all regulatory activities (reviews and decisions) associated with biologically-based pesticides and emerging technologies. In 1979, the US EPA published a policy statement on “biorational” pesticides (biopesticides) that formally recognized microbial pesticides as having unique characteristics that set them apart from chemical pesticides and committed US EPA to developing a set of data requirements and testing guidelines for microbial pesticides [91]. These guidelines, referred to at the time as “Subdivision M” guidelines, were initially published in 1982 [92], followed by official publication of data requirements for biopesticides in 1984 in the US Code of Federal Regulations. These publications set forth the maximum hazard dose approach to testing and tiered testing strategy that is in place today. As more information was gathered on microbial pesticides, further revisions of the testing guidelines and data requirements were made in 1989 [93] and 1994 [94], respectively. The guidelines and data requirements were published in their current states in 1996 [95] and 2007 [96, 97], respectively. Throughout the development of the US EPA’s guidelines, the agency has recognized the need for flexibility in testing protocols to account for numerous requirements associated with both the microbial test substance and relevant test organisms.

### Data requirements in EU and US

The data requirements for the EU and the US EPA are highlighted here, as these regulatory processes are the most followed in regions where local regulatory regulations for biopesticide products are not available or vague. However, recently many countries around the world have implemented similar requirements to the EU and US EPA.

#### Data requirements for active substance

For both US and EU, the data requirements for microorganisms as active substances are structured in a step-wise approach (see Table 1: US and EU data requirements for microorganisms as active substance). However, these data requirements differ in the endpoint of toxicity. Whereas in the US the endpoint of toxicity is related to the active substance; in the EU the toxicity is related only to metabolites but not to the active substance itself.

**Table 1:** US and EU data requirements for microorganisms as active substance

**US Data Requirements**	OPPTS / OECD Guideline / Other
***Tier 1***	
Acute Oral Toxicity/Pathogenicity^a^	885.3050
Acute Dermal Toxicity/Pathogenicity^a^	885.3100
Acute Pulmonary Toxicity/Pathogenicity^a^	885.3150
Acute Injection Toxicity/Pathogenicity^a^	885.3200
Hypersensitivity Incidents (reported)	885.3400
Cell Culture^b^	885.3500
Acute Oral Toxicity^c^	870.1100 / 425
Acute Dermal Toxicity^c^	870.1200 / 402
Acute Inhalation Toxicity^c^	870.1300 / 403
Acute Eye Irritation	870.2400 / 405
Acute Dermal Irritation	870.2500 / 404
Dermal Sensitization	no study required due to absence of valid study due to default label statement
***Tier 2***	
Acute Toxicology	885.3550^d^
Subchronic Toxicity/Pathogenicity	885.3600
***Tier 3***	
Reproductive/Fertility Effects	885.3650
Carcinogenicity^e^	870.4200
Immunotoxicity^e^	870.7800
Infectivity/Pathogenicity Analysis	885.3000^f^
**EU Data Requirements**	OPPTS / OECD Guideline / Other
***Basic infectivity and pathogenicity studies***	
Oral Infectivity/Pathogenicity^g^	885.3050
Intratracheal/intranasal Infectivity/Pathogenicity^g^	885.3150
Intravenous, intraperitoneal or subcutaneous single exposure^g^	885.3200
Hypersensitivity Incidents (reported)	885.3400
Cell Culture	885.3500
***Specific infectivity and pathogenicity***	study depending on finding which requires further investigation
Subchronic Toxicity/Pathogenicity	885.3600
Reproductive/Fertility Effects	885.3650
***Information and toxicity studies on metabolites***	SANCO/2020/12258
Dermal Sensitization	no study required due to absence of valid study; Regulation (EU) 283/2013 provides for obligation to consider MO as potential sensitizers until a validated test is available and the possible absence of sensitization potential is demonstrated on a case-by-case basis

^a^In some instances, waivers may be acceptable to the authorities and will be considered on a case-by-case basis

^b^Required for viruses only

^c^Required if route of administration is not covered in pathogenicity studies

^d^Route or routes of exposure based on toxicity was observed in pathogenicity studies from Tier I. (OPPTS 870.1100, 870.1200, 870.1300/885.3150)

^e^Considerations for virus is active substance

^f^See guideline for additional requirements and considerations for Tier 3

^g^required unless the applicant can demonstrate absence of infectivity and pathogenicity based on a weight of evidence approach

The first step/tier requires basic pathogenicity (and toxicity) information on the microbe. This safety data contains information on the assessment on potential infectivity and pathogenicity (and toxicity) of the microorganism. Pathogenicity studies for microbial active substances are only required if the applicant cannot use a weight of evidence approach to demonstrate absence of infectivity and pathogenicity. In case pathogenicity studies have to be conducted, they can be performed via the oral, intratracheal/intranasal, intravenous/intraperitoneal or dermal routes of exposure. In the US, additionally skin and eye irritation studies are required to evaluate potential irritancy of the microbe. Currently, no validated tests are available for both irritating endpoints which may to be used in the US and the EU. Hypersensitivity data/medical data are reported if there is any information or medical surveillance information on manufacturing plant personnel, information on sensitization/allergenicity, and direct exposure observations. For viruses, cell culture studies may be also required. Additional steps/tiers include studies to further evaluate the adverse effects observed in the Tier I studies, which includes specific infectivity and pathogenicity studies on the microorganism, and potential information and toxicity studies on metabolites. The proposed test guidelines in the EU and the US are according to the Office of Prevention, Pesticides, and Toxic Substances (OPPTS) 885 series and respective OECD and other guidance documents.

#### Data requirements for formulation

Formulations can contain one or more active substances (technical grade active ingredients = TGAI), co-formulants, safeners and synergists. For ease, only the singular form of the active substance will be used: formulations with a microorganism as active substance.

In the EU, the concrete statement of the data requirements for formulations is outlined in Regulation (EU) No 284/2013 [87]. This regulation addresses formulations with chemicals as active substance in Part A, and formulations with microorganism as active substance in Part B. The Part B of this regulation has been revised. The new proposal for these data requirements of Regulation (EU) No 284/2013 Part B, entered into force in November 2022, is structured in a three-step approach to support the 3-R principle for refinement, reduction and replacement of animal use [98] and represent a responsible approach to consider animal welfare. First, medical data may be provided. This comprises any available information on possible adverse effects to human health, including sensitization and allergenic response of humans exposed to the formulation observed during development and manufacturing. Second, the weight of evidence approach may be applied. This approach may be defined by providing information to determine the potential toxicity of the formulation from any other reliable sources [e.g. Integrated Approach to Testing and Assessment – IATA, acute toxicity estimates of formulations in accordance with the Regulation (EC) No 1272/2008 [99], read-across data from similar preparations, or New Approach Methods (NAMs)] to demonstrate that no toxic effects are to be expected or that some toxicity is expected, and then the results can inform further investigations. In the last step, available information derived by toxicological studies may be used to classify the formulation in accordance with Regulation (EC) No 1272/2008 with regard to toxicity to humans.

In the US, the data requirements for the formulation containing microorganisms as active substances are described in Title 40, Chapter I, Subchapter E, Part 158, Subpart V, § 158.2120 of the Code of Federal Regulations [100].

In both regulations, the data requirements are outlined for the following toxicological endpoints: acute oral toxicity, acute dermal toxicity, acute inhalation toxicity, skin irritation, and eye irritation and skin sensitization. Unless no medical or no other information for application of the weight of evidence approach can be provided that allows an assessment of the possible acute toxicological endpoint of the formulation, a test or alternatives may be provided (see below and Table 2: US and EU data requirements for microbial products). Challenges associated with the use of or adaption of current NAMs for microbial pesticides are discussed below; in all cases the validation of NAMs has not included microbial pesticides. Proposed test guidelines in the EU are according to OECD guidelines and in the US are according to the OPPTS 870 series, or equivalent OECD guidelines. The mentioned guidelines describe in vivo testing procedure unless it is stated otherwise.

**Table 2:** US and EU data requirements for microbial products

	OPPTS / OECD Guideline / Other	
**Data Requirements**	**US**	**EU**
Acute Oral Toxicity	870.1100 / 425	420, 423
Acute Dermal Toxicity	870.1200 / 402	402
Acute Inhalation Toxicity	870.1300 / 403	403, 436
Acute Dermal Irritation	870.2500 / 404	404 in vitro tests acc. to OECD TG 430, 431, 435, 439 IATA for Skin Corrosion and Irritation, No. 203
Acute Eye Irritation	870.2400 / 405	405 in vitro tests acc. to OECD TG 437, 438, 460, 491, 492 IATA for Serious Eye Damage and Eye Irritation, No. 263
Dermal Sensitization	870.2600^a^ / 429^a^	406, 429, 497 in vitro tests acc. to 442A, 442B, 442C, 442D, 442E
Additional toxicity information	submitted case by case	based on expert judgement case-by-case acc. to Reg 284/2013

^a^Due to default label statement, dermal sensitization studies are not run for all TGAI and formulated products. Their applicability for microbial products is currently under discussion

### Comparison of regulatory procedure and data requirements to chemical pesticides

This chapter provides a brief overview of the comparison of microbial and chemical pesticides in respect of general regulatory procedure, data requirements for the formulation and the active substance. Furthermore, differences between the EU and the US were shown if applicable.

The two-step regulatory procedure – at first the active substance approval and then the authorization of the plant protection product(s) – is identical for the chemical and microbial active substances and the plant protection products (PPP) containing them. This two-step regulatory procedure is conducted in the EU and US as well.

The data requirements for the formulation differ slightly between PPP containing chemical and microbial active substances. In addition to the six tests required for PPP containing chemical active substances (tests on acute oral, dermal, and inhalation toxicity; test on skin irritation/corrosion potential, test on eye irritating/corrosion potential and on skin sensitization) information on medical data have to be considered as well due to their sensitizing potential and the possible allergic response of humans exposed to the product. However, data on dermal absorption are not required for formulations containing microorganisms as active substance as it is assumed that the microorganism does not penetrate human skin. It may be noted that components of the formulation other than the active substance may possibly alter the penetration process and therefore may also allow microorganisms to penetrate through human skin [87, 88]. The applicability of skin sensitization test(s) for microbial products is currently under discussion in the EU; default labelling is therefore required. This is similar to the US, where due to default label statement dermal sensitization studies are not run for all TGAI and formulated products.

The data requirements for the active substance, concerning human health, are different whether it is a chemical or a microorganism. For chemical active substances toxicological and metabolism studies are required. These additional studies for chemical active substances include studies on absorption, distribution, metabolism and excretion in mammals; acute, short-term and long-term toxicity studies including carcinogenicity; genotoxicity testing, reproductive/developmental toxicity, neurotoxicity studies and other toxicological studies (e.g., evaluations of metabolites, potential endocrine disrupting properties). Information on medical data is also required [85]. For microbial active substances three main differences are the following: 1) a literature review of relevant and reliable information and the weight of evidence approach are considered (For chemical active substances toxicological studies are required); 2) if no relevant information exists studies on acute infectivity and pathogenicity are conducted (not long-term toxicity and carcinogenicity studies for chemical actives [85, 86]); 3) secondary metabolites produced by the microorganism are evaluated and additional toxicological studies may be necessary. A guidance document was developed to determine what is considered “of toxicological relevance” of microbial secondary metabolites [101]. The difference between the EU und the US is the approach of using the information from the data requirements. In the EU the “weight of evidence” approach is taken first, then testing is performed. In the US, the “weight of evidence” approach is used more in tandem with testing and other information.

## Challenges associated with data requirements endpoints

### Acute infectivity and pathogenicity of microbial active substances

The data requirements for testing of microorganisms require the assessment of the infectivity and pathogenicity via up to four routes: oral, dermal, respiratory (usually intratracheal/intranasal), and injection (usually intravenous, intraperitoneal or subcutaneous). US EPA OPPTS guidelines are normally employed for each of the four study routes: 885.3050, 885.3100, 885.3150, and 885.3200, respectively [102–105]. The purpose of the acute oral/dermal/pulmonary/injection studies are to provide initial information on the infectivity and pathogenicity of a microorganism using a single dose exposure and an adequate post-exposure observation period. Principles of the test method are to administer the MPCA orally by gavage/ dermal/ by intranasal or via intratracheal routes/ by intravenous, intraperitoneal or subcutaneous injection in a single high dose to experimental animals. Subsequent observations of effects and deaths are made and rate of clearance of the microorganism is estimated over a period of 21 days (in the EU the length of the observation period is decided based on the biological properties of the microorganism). Animals are sacrificed and necropsied throughout the 21 days to determine potential clearance and infectivity in targeted organs. For testing on acute pathogenicity and infectivity of microorganisms, currently no non-animal testing methods are available, although proposals and discussions are on-going at the OECD level to consider using whole genome sequencing or other alternative strategies (e.g., bridging).

To consider reducing the number of pathogenicity studies, a two-step approach may be proposed following the 3-R principles [98]. Therefore, the proposal would be to start with the first study on infectivity/pathogenicity study in rats for the most relevant route of exposure of the microbe. Depending on the results, further animal testing might not be scientifically justified. This step may be considered as confirmation of results of literature search which can further substantiate the absence of any indications for an infective/pathogenic effect. The inability of growth at or around body temperatures could also support an additional argumentation for not performing further animal testing. If there are indications of adverse effects and/or lack of sufficient clearance, a second route of exposure (step II) could be considered. This two-step approach might be useful to lower the number of tests required for assessing the safety of microorganisms.

With this proposal the question arises: which route of administration should be used for step I and step II? Considering, for example, human non-dietary (mixer/loader/applicator) exposure during and after application of the microbial pesticides, the oral route seems to be not the most relevant pathway as the applied formulations were not orally ingested if used appropriately; in respect of dietary exposure, there are likely no viable residues on edible plant parts. Furthermore, many microorganisms might already be inactivated by the gastrointestinal passage if ingested. The injection pathway (usually intravenous) may be also discussed for less relevant administration route. It may seem not fit to the common use of plant protection products, as no exposure would occur via this route, however, this route of administration bypasses the gut. Therefore, the respiratory pathway may be fit to cover expected human exposure for mixer/loader/applicators. However, dermal exposure can also be considered as the primary pathway for non-dietary exposure and therefore may also be considered in this approach. The issue for a dermal exposure route of administration is that microorganisms, in general, do not cross the skin barrier due to their size and properties [106]. In conclusion, considering the appropriate route of administration to confirm the lack of pathogenicity and infectivity with the aim to reduce the animal tests could be to evaluate up to two routes of administration, and should be considered on a case-by-case basis.

To reduce the number of animals that need to be tested to evaluate the safety of microbial biopesticides, the reduction of treatment groups based on the results obtained in Step I might also be reasonable. Therefore, one full infectivity/pathogenicity study as Step I proposed could be conducted and then another study, could be performed as necessary. A proposal to further support reducing animal testing might be the waiving all specific routes of administration based on other scientifically relevant information discussed with the authorities at a pre-submission meeting.

It may be noted in general, that pathogenicity testing may be considered as crucial point for the assessment of microorganisms due to their host range specificity. Test animals may fall without this host range (while humans fall within). For future discussions the following points need to be clarified: is testing in animal models required to address pathogenicity, and which animal model may be the best fit for purpose.

### Genotoxicity of microbial active substances

In general, genotoxicity testing answers a very different question than a subchronic or developmental toxicity study, and is a basic requirement for chemical pesticides. The possibility of the inclusion of this basic requirement for microbials will be discussed in the following.

The testing also follows a tiered safety testing strategy. No hazards identified means that no further testing is required. On the other hand: if hazards are identified (e.g., by literature, WGS, animal studies) additional testing could be considered.

In practice, the Ames test (OECD 471, [107]) is the most frequently used test for providing data for this endpoint for microorganisms [108] even though there is no specific guideline for assessing genotoxicity. However, genotoxicity testing by using the Ames test demonstrates methodological challenges when testing MPCAs. The Ames test is based on the detection of gene mutations induced by the tested compound when amino-acid requiring strains of *Salmonella typhimurium* grow on a minimal medium after exposure to the test substance. This test may not be applicable if the test substance is a living organism that might have antimicrobial properties, but it might allow for testing of secondary metabolites produced by the microorganism if whole genome sequencing has not assessed genotoxicity potential. The main issues around the use of the Ames test for microbial active substances relate to (1) growth of the MPCA on the agar plates used to culture the treated bacterial strains and (2) components of the microbial active substance acting as a food source for the bacterial strains used in the assay. It is possible to avoid (1) by using inactivated microbial active substance or lysed cells; and (2) through the use of modified protocols e.g., ‘treat and plate’ methodology. It needs to be considered that the inactivated microbial active substance might be applicable for the test system but does not represent the substance used in the applied formulation.

Additionally, there are some limitations as the Ames test only detects gene mutations, but not chromosome and genome mutations. Moreover, it is done with a procaryotic organism and not on a eukaryotic mammalian system. Other guideline studies are available to cover chromosome and genome mutations (especially the micronucleus assay) with mammalian systems. Nevertheless, among the OECD guidelines available for the investigation of genotoxicity and mutagenicity, the same challenges for testing a living organism may be faced when working on eukaryotic in vitro cell systems.

Nevertheless, some microbial organisms used as biopesticides can produce toxins, as for example mycotoxins produced by *Beauveria bassiana* (see the EFSA conclusion [90]), the mutagenic hazard cannot be excluded and this endpoint should not be disregarded. However, for some known produced metabolites, it is possible to refer to data already published in the literature or to the conclusions produced by any regulatory agency. The work of EFSA [90] also highlighted that the genotoxic potential of beauvericin, a mycotoxin which can be produced by *Beauveria bassiana*, cannot be excluded. The genotoxic potential is based on positive in vitro chromosomal aberrations and micronucleus test and equivocal in vivo data [90]. In addition, quantitative structure–activity relationship models are not appropriate for MPCAs, as microorganisms produce hundreds or more secondary metabolites. Thus, testing for biopesticides should cover the range of possible mutations in genes, chromosomes and genome: how to do this is yet to be fully evaluated.

### NAMs for acute toxicity for microbial pesticide formulations

In this chapter the currently proposed or already harmonized NAMs for chemical pesticides are presented and discussed for the proposed use for microbial pesticide formulations.

#### Acute oral toxicity

If data or information are not available to conclude on the acute oral toxicological potential, or if an acute oral pathogenicity study is not conducted for the microbe, a test for acute oral toxicity may be carried out in accordance to Regulation (EC) No 440/2008 and according to OECD and US EPA guidelines (OECD 420, 423; 870.1100) [109–111]. These guidelines involve the administration of a single oral dose of test substance to fasted healthy young adult rodents, usually of one sex, by oral gavage, observations for 14 days after dosing, recording of body weight, signs of toxicity, death and necropsy including notation of gross lesions of all animals [112].

At present, the acute toxicity estimate (ATE) calculation method is discussed as an alternative for in vivo acute oral toxicity studies. This calculation method is in accordance with the Regulation (EC) No 1272/2008 [99], and is required, or at least accepted, in some regions. The ATE calculation method is based on the additivity principle, which considers acute toxicity estimate values for all relevant ingredients. Several authors have reported the use of ATE calculation method for predicting Classification, Labeling and Packaging (CLP) and US EPA toxicity categories for conventional agrochemical formulations with a varying degree of accuracy, which makes it difficult to have full confidence in the ATE calculation method. Hamm et al. showed that the ATE calculation method has a 52% correct prediction of US EPA categories for agrochemical formulations (*n* = 620) [113]. A lower value for prediction was provided by Van Cott in 2018 by showing a 43% accuracy in prediction of US EPA categories (*n* = 210) [114]. A much higher value for prediction was demonstrated by Corvaro et al., with 75% correct prediction of US EPA categories (*n* = 199) [115]. In these reports, there is a trend of the ATE calculation method underpredicting the acute oral toxicity category for more toxic agrochemical formulations, though these are often underestimated in the datasets: US EPA category I and II formulations represent 0% and 7.5%, respectively, of the formulations in the dataset of Covaro et al. [115], and 0.5% and 35%, respectively, of the formulations in the dataset of Van Cott et al. [114]. In the evaluation by Kurth et al., the ATE calculation method was compared to the in vivo derived CLP acute oral toxicity categories, and again the ATE calculation method underestimated the in vivo toxicity in approximately 40% of classified formulations (*n* = 95), where the misclassification as associated with the failure to distinguish between CLP category 4 and no classification [116]. There were no formulations in this dataset for the more toxic CLP categories, namely categories 1, 2, and 3. This underestimation makes it difficult to use the ATE method confidently in a human health risk assessment without additional relevant scientific data. Additionally, a more rigorous evaluation of the predictive capability of the ATE calculation method for the more toxic categories is necessary to build confidence in its utility in risk assessment. Therefore, for considering the ATE calculation method as an appropriate alternative for in vivo testing further evaluation of the method and the toxicological data set for all components of the formulation – TGAI and co-formulants—is needed in the author’s opinion.

The main endpoint of the acute oral toxicity study is mortality or impending death and the check for clinical symptoms and macroscopic and microscopic analysis of organs. A possible approach, but not according to REACH, is the in vitro cytotoxicity test method, where it was possible to predict the oral acute toxicity [117]. The replacement of in vivo acute oral toxicity studies by in vitro cytotoxicity methods was discussed before [118–120]. Unknown mechanisms leading to the observed acute oral toxicity in vivo and the lack of capability to assess vital parameters in vitro were pointed out as the major aspects challenging the development of alternative approaches [119]. Based on computer simulations for reference substances tested in the study, it was recommended that a specific test of cytotoxicity may be used as part of weight of evidence approach for selection of the starting doses for rodent acute oral toxicity testing. This tiered approach seems to have the potential to reduce the number of animals [121]. However, in a critical review from Schrage et al. in 2011 [122], the use of cytotoxicity data for selecting an in vivo starting dose was evaluated. The results from the in vitro Balb/c 3T3 NRU cytotoxicity test conducted according to the Interagency Coordinating Committee on the Validation of Alternative Methods (ICCVAM) 2006 report [123] were compared to the results from the in vivo test according to OECD 423 [110], for a total of 203 substances (16 substances from Halle Register 2003 [117] and 187 substances including chemicals, agrochemicals and formulations thereof [122]). The cytotoxicity assay demonstrated a good prediction for weakly toxic substances (74% correctly identified as CLP category 4), but not for the other CLP categories. Therefore, there was a low overall concordance of 35% in total [122]. In predicting a starting dose that matches the in vivo classification, the use of data from the cytotoxicity test was comparable to the use of a default starting dose of 300 mg/kg bw (58% and 50%, respectively), but far inferior compared to the prediction of an experienced toxicologist (95%). Furthermore, the selection of the starting dose based on the cytotoxicity test would have result in higher numbers of animals per test than the selection based on expert judgment. The authors conclude that the prediction of a starting dose by using cytotoxicity data would not have contributed to either refinement or reduction in acute oral toxicity testing in vivo [122].

The value of this aggregated data on agrochemical formulations demonstrates some accurate and some inaccurate calculations of synthetic chemistries can that make it difficult to rely on the ATE calculation method for microbial formulations without additional toxicity information on the microbe, or without specific evaluation of this calculation method for microbial formulations. However, for microbial formulations, the co-formulants are rarely added in high concentrations, are usually preservatives with low toxicity, and do not interact with the microbial, making this ATE calculation method a part of weight of evidence approach to consider for reducing animal use. Nevertheless, the use of ATE calculations may provide a means of estimating the acute oral toxicity where the microorganism active substance and other co-formulants are known to be of low toxicity, and/or are of low concentration in the formulation.

#### Acute dermal toxicity

To assess acute dermal toxicity, an in vivo test may be carried out in the EU in accordance to Regulation (EC) No 440/2008 and according to OECD guidelines (OECD 402) [124] and in the US according to OPPTS 870.1200 [125] intended the single application of MPCAs in each formulation to be tested in a single high dose of microorganism to the skin of experimental animals.

The intact skin provides a physical barrier which microorganism cannot enter due to their size, and the skin produces chemical compounds which are either microbiocidal or microbiostatic [106]. Despite this, in many cases it is not the microorganism per se that induces skin reactions, but proteins, glycoproteins or secondary metabolites of the microorganisms [126]. Gloves may be required in some regions due to the precautionary labelling. They are required for the operators/mixer/loaders when handling and applying any biopesticide which would protect the operator from the majority of the potential dermal exposure [127]. However, the use of gloves is not a common practice in certain geographies. A cross-sectional study with 600 paddy-field workers in India revealed that 85% of workers did not wear any kind of personal protective equipment (PPE) [128], and therefore the assessment of dermal toxicity is important to ensure the safe use and application of microbial products. Additionally, the potential presence of small skin lesions, which can be common due to the physical nature of the agricultural workers’ typical tasks, is a factor that could increase dermal exposure in agricultural workers. Taking together that farmers may not have access or be accustomed to wearing PPE for skin exposure, and the potential to have a compromised skin barrier, it may not be surprising that synthetic and microbial pesticides may cause adverse effects to the agricultural workers’ skin [129]. Therefore, understanding the acute dermal hazards associated with a formulation seems necessary.

In vitro testing methods for acute dermal toxicity in agrochemicals are currently not available. However, the in vivo test for acute dermal toxicity has at least the potential to reduce animal testing. Currently at least three or five animals of each sex are required per study [124]. It may be possible to limit the group size to three or five animals of a single sex [121, 124], preferably the most sensitive sex. Kurth et al. demonstrated that in the few instances that a conventional pesticide formulation in their data set was classified for acute dermal toxicity, it was usually based on and in vivo test with the active ingredient [116]; however this type of evaluation has not yet been conducted for microbial pesticides. The ATE calculation method may have the potential to be used to assess acute dermal toxicity considering its limitations discussed above. Covaro et al. reported a 92.1% correlation of the ATE calculation method with in vivo derived US EPA categories (*n* = 179 formulations), and a 99.5% correlation with in vivo derived CLP categories (*n* = 207 formulations) [115]. Van Cott et al. reported a correlation of the ATE calculation method of 48% with CLP classifications and 36% with US EPA classification for 31 formulations [114]. As with the acute oral toxicity datasets described above, the more toxic categories were underrepresented in the aforementioned datasets, limiting the evaluation of the predictive capability of the ATE calculation method for the more toxic US EPA or CLP categories for synthetic chemistries. However, similar to the evaluation for oral toxicity of microbial formulations, utilizing the ATE calculation method to estimate the acute dermal toxicity for biopesticide formulations could be a part of weight of evidence approach, when utilized when acute dermal toxicity data on the microorganism active substance and co-formulants are available.

One option to reduce the reliance on in vivo acute dermal toxicity tests might be through the use of waivers. The US EPA conducted a comparison of paired the acute oral and dermal LD_50_ from rat studies for 592 formulated pesticide products. For 57% of the formulations the results of the oral and dermal acute studies resulted in the same toxicity category, and for 38% the acute oral toxicity study resulted in a more toxic category. Therefore for 95% of the formulations the acute dermal toxicity study had no impact on the classification of on PPE requirements. The US EPA began accepting waivers for the acute dermal toxicity study for formulations in 2016 [130], and will accept them for biological pesticide formulations.

#### Acute inhalation toxicity

The waiving of an acute inhalation toxicity study of a formulation is not possible if the formulation is used with fogging equipment, used as a smoke generating formulation, used as a vapour releasing preparation, is applied from aircraft in cases where inhalation exposure is relevant (broadcast air-assisted sprayer), used as an aerosol, used as a powder containing a significant proportion of particles of diameter < 50 µm (> 1% on a weight basis), used in a manner which generates a significant proportion of particles or droplets of diameter < 50 µm (> 1% on a weight basis), or containing a volatile component at greater than 10%, according to the Regulation (EU) No 284/2013 [87]. The test for acute inhalation toxicity may be carried out in accordance to Regulation (EC) No 440/2008 [131] and according to OECD guidelines (OECD 403, 436) [132, 133] in the EU. In the US, the testing of acute inhalation toxicity in accordance to OPPTS 870.1300 is required [134].

The ATE calculation method for mixtures is discussed as one alternative for acute inhalation toxicity tests in vivo. This method is in accordance with the Regulation (EC) No 1272/2008 [99]. The ATE method was evaluated in a dataset of agrochemical synthetic pesticide formulations containing 7.3% of CLP classified formulations. It has been shown that the calculation method is able to predict in vivo derived classification accurately in 94.3% of the cases, though this is of limited value since the vast majority (114 of 123) of the formulations were not classified [115]. The same dataset was evaluated for the prediction of US EPA categories, and there the ATE calculation method accurately predicted 96.7% of the formulations [115]. Van Cott et al. report a correlation of the ATE calculation method of 61% with CLP in vivo classifications and 73% with US EPA in vivo classifications for 128 formulations; for this evaluation of the ATE calculation method, the data set was again weighed heavily towards the less toxic CLP and US EPA categories [114]. Kurth et al., report a correlation of approximately 45% of PPPs classified for inhalation toxicity compared to the predictions made by ATE method [116]. However, this ATE method for inhalation toxicity of microbial pesticide formulations could be a part of weight of evidence approach to use for microbial pesticide formulations due to the reasons mentioned above.

Harmonized non-in vivo testing methods for acute inhalation toxicity are currently not available. In respect of the 3-R principle, within some revision of the OECD guideline 403 and 436, lowering the number of animals and incorporating scientific advancements were considered [132, 133]. The report of a FRAME workshop in 2009 provided a number of in vitro tests which may be applicable for testing on inhalation toxicity [135]. The in vitro tests can be distinguished into two cell culture growing systems: one uses submerged cells: these are cultures where cells are grown in liquid; the other uses air–liquid interface (ALI), where cells are growing in direct contact with air. By evaluating liquid submerged cells and cells grown in ALI for a number of toxicity markers and morphology, the ALI cultures showed a permeability and theoretical pore size that were more representative of the in vivo respiratory epithelium than were exhibited by the submerged cells. In 2016, a workshop in Paris, France was organized to discuss ALI cell culture models which may potentially be used to assess inhalation toxicology endpoints [136]. The participants concluded the same as stated above: ALI systems are more relevant to the in vivo situation than any other currently available in vitro approach based on submerged cell cultures [136]. A perspective article from 2020 describes in vitro alternatives to acute inhalation toxicity studies in animal models [137]. The authors pointed out the currently poor translational rate of current in vitro alternatives into regulator-approved methods. They also present the authors’ perspective on how it may be possible to overcome the current challenges in validating in vitro alternatives for the successful replacement of animal-based inhalation toxicity testing studies. At the end, the authors concluded that in vitro acute inhalation toxicity testing should use exclusively cell cultures in ALI conditions as this mimics the most realistic way of exposure [137].

Recently, a submerged 2-D lung cell culture model was considered to correctly predict in vivo acute inhalation toxicity for substances with a water solubility of ≥ 1 g/L in semi-volatile organic compounds and non-volatile organic compounds [138]. Other systems have been also pre-validated, for example the EpiAirway™ In vitro human airway model [139]. This commercially available in vitro organotypic model of human mucociliary airway epithelium were exposed to 59 test chemicals for three hours directly with immediate postexposure viability testing. Sensitivities of 87.5%–100% and specificities of 56%–89% were reported [139]. Further alternative approaches for acute inhalation toxicity testing for chemicals were discussed on an international workshop in 2016 [140].

Although in vitro models using cultured cells may be validated for the assessment of local effects of synthetic pesticide test materials, one limitation is that they do not assess systemic toxicity (i.e., toxicity in organ systems beyond the lungs) which may be caused by components of the microbial active substance (e.g., toxic metabolites). Considering the challenges with accuracy and predictivity for synthetic pesticides, the utility of alternative studies to determine inhalation toxicity including microbials is not yet well understood, and the utilized ATE calculation method could be a promising part of a weight of evidence approach when acute inhalation toxicity data on the microorganism active substance and co-formulants are available.

#### Irritation

##### Skin irritation

Considering the endpoint of skin irritation/corrosion, at first the IATA approach for Skin Corrosion and Irritation could be used [141] an in vivo test for skin irritation according to the Draize method shall only be conducted if in vitro methods are not applicable for the substance (gases or aerosols) or if in vitro studies are not enough for classification [142]. The in vivo test may be carried out in accordance with the most appropriate guidelines (OECD 404, OPPT 870.2500) [143, 144].

The GHS or CLP additivity calculation method could also be considered as an alternative to in vivo testing. A comparison of in vivo data and calculation-derived classification for a set of CLP category 2 synthetic chemistry formulations (*n* = 32) revealed 69% accuracy of the additivity method [116]. However, the additivity method underestimated and overestimated the skin irritation potential for 22% and 9%, respectively, of the CLP category 2 in vivo classified formulations [116].

In the last decade, three OECD-compliant in vitro tests assessing the skin corrosivity have been established: OECD 430 (Transcutaneous Electrical Resistance Test) [145], OECD 431 (Human Skin Model Test) [146], and OECD 435 (In vitro Membrane Barrier Test) [147]. A negative result of these in vitro tests is usually followed up with a test for skin irritation, as these in vitro tests only inform on skin corrosivity and not on their skin irritant potential. The Reconstructed Human Epidermis Test Method (OECD 439) [148] was validated for assessing skin irritancy potential. This test provides an in vitro procedure that may be used for the hazard identification of irritant chemicals and can also be used to identify non-classified chemicals. It has to be noted, that these in vitro tests may not be applicable for formulations, as the correlation between the results gained in the in vitro test and the CLP classification resulted in only 44% sensitivity, 60% specificity and 54% accuracy for a dataset of 65 formulations [149].

The applicability of these in vitro tests for microbial pesticide testing may be evaluated in the alternative models depending upon the study requirements. Several issues have been identified when discussing the utility of alternative studies for microbial pesticides including: soluble test material is needed for multiple assays; test materials are required to have a known concentration; potential interference of microbial products or large proteins with detection methods like fluorescence; and lack of true microbial positive or negative controls, which could help interpret study results. A promising outlook in direction of study applicability provides the data reported by Nikodinoska and coauthors [150]. They tested in vitro skin irritation assays (OECD 439 and a modified OECD 439) with six lactic acid bacteria strains. Modifications of the main experiment according to OECD 439 [148] were made during the test item removal step. In the recovery phase the treated tissues were incubated in medium supplemented with 8–10 µg/mL streptomycin [150]. Data show that the test items are not suitable for the OECD 439 protocol, but may be suitable for the modified protocol. The authors describe the validity of the modified test, and conclude that the test items were considered as not irritating to skin by using the modified OECD 439. Furthermore, the addition of an antibiotic during the recovery phase did not influence tissue viability. This initial attempt at validation of this testing strategy needs further investigation to understand the utility with additional microbial strains (multiple genus) to come to a conclusion on whether this alternative in vitro testing method is applicable to these test materials.

In conclusion, scientific progress has been made towards answering applicability aspects of in vitro assays for microbials and the utilized GHS/CLP additivity method may be a part of a weight of evidence approach, when skin irritation data on the microorganism active substance and co-formulants are available.

##### Eye irritation

If a test for eye irritation is required, it is necessary to carry it out with the most appropriate guidelines as a first choice with the IATA approach for Serious Eye Damage and Eye Irritation (OECD 263) [151] and at last resort with in vivo testing (OECD 405) [152] in the EU. In the US, the testing of eye irritation in accordance with OPPTS 870.2400 or OECD 405 is required [153].

Evaluation of synthetic agrochemical formulations using the GHS or CLP additivity calculation method as a possible alternative to in vivo testing showed an accuracy of 56% for the formulations compared to the results achieved by in vivo testing was obtained based on CLP category 1 and 2 classified formulations (*n* = 90). Contrary to the results obtained for skin irritation, 6% of the formulations were still underestimated in respect of their eye irritation potential and almost 40% were overestimated. Due to the high rate false-positive results, the CLP additivity method is predisposed to overestimating the potential for eye damage [116]. This dataset does not include any microbial formulations, which are comprised of many proteins, glycoproteins or secondary metabolites of the microorganisms, and not just a single active ingredient to evaluate.

There are several validated and OECD-compliant in vitro tests currently available. The organotypic Bovine Corneal Opacity and Permeability Test (OECD 437) [154] and Isolated Chicken Eye Test (OECD 438) [155], as well as the in vitro Short Time Exposure In Vitro Test Method (OECD 491) [156] may identify compounds inducing serious eye damage (Category 1) and substances not requiring classification for eye irritation or serious eye damage (No category). The Fluorescein Leakage Test Method (OECD 460) [157] identifies serious eye damage (Category 1), therefore, this test is recommended for use as part of tiered testing strategy for regulatory classification and labelling. For the identification of chemicals not requiring classification and labelling for eye irritation or serious eye damage the Reconstructed Human Cornea-like Epithelium (RhCE) test method (OECD 492) [158] may be appropriate. The OECD test guideline 492B [159] describes a procedure allowing the identification substances and formulations in all Globally Harmonized System of Classification and Labelling of Chemicals (GHS) eye hazard categories, i.e. those not requiring classification (No Category), requiring classification for eye irritation (Category 2) and requiring serious eye damage classification (Category 1) [160]. Defined approaches are described in the OECD test guideline 467 proposing the combination of data generated by in vitro methods to determine eye hazard potential according to the hazard classes of the GHS [161].

However, this set of in vitro tests has its limitations for agrochemical formulations and were reported by Kolle and coauthors [162]. Kolle and coauthors observed that the organotypic Bovine Corneal Opacity and Permeability Test (OECD 437) [154] shows low specificity for non-severe eye irritant agrochemical formulations, whereas the Isolated Chicken Eye Test (OECD 438) [155] shows under-prediction for severe eye irritant agrochemical formulations [162]. Further investigations revealed that the EpiOcular-Eye Irritation Test, which follows the OECD 492 test guideline, is currently the best in vitro method for the prediction of the eye irritation potential of liquid agrochemical formulations [163].

As for the skin irritation also for eye irritation, Nikodinoska and coauthors provide an interesting initial investigation in the study of the applicability of in vitro assays for microbials [150]. The authors investigated the applicability of the RhCE test method (OECD 492) [158] and the in vitro Short Time Exposure In Vitro Test Method (OECD 491) [156] and the use of six lactic acid bacteria strains. Data show that the test items are not suitable for the OECD 492, but may be used in the test described in OECD 491. They conclude that the test items were considered as not irritating to the eye as both tested concentrations of 0.05 and 5% (w/w) did not induce cell viability reduction below 70%. However, it is unclear how other microbial biopesticides would perform in these assays (i.e. whether they would also face the same, rather significant technical challenges, and wider testing is needed to provide information on the applicability domain).

Data from Nikodinoska and coauthors [150] describe the scientific progress towards answering applicability aspects of in vitro assays for microbials. The appropriateness of non-animal tests for the evaluation of microbial pesticide serious eye damage may be further explored by engaging with stakeholders to validate the alternative studies and explore the utility of the GHS additivity method with microbials to determine whether these could be reliable alternatives to the traditional in vivo test.

#### Sensitization

For microorganisms, sensitization is the most challenging endpoint, as in the EU and US the active substance itself is considered as “potential sensitizer” according to Commission Regulation (EU) 2022/1439 amending Regulation (EU) 283/2013 [85, 86] and US 40 CFR Part 158, Subpart V [100], and currently no validated test method exists to confirm or disprove this statement.

##### Skin sensitization

Skin sensitization and microorganisms are challenging as the currently used strategies might help for the moment but have plenty of room for improvement. Considering the formulation and the endpoint of skin sensitization, numerous in vivo and in vitro OECD-conforming methods already exist.

The test methods may be grouped by their outcome related to specific phases and key events in the skin sensitization process. This process involves two phases, an induction and elicitation phase [164–166]. Skin sensitization is induced when a susceptible individual is exposed topically to the potential allergen. To elicit a cutaneous immune response, a chemical must gain access to the viable epidermis [165]. In the elicitation phase, the T-cells are activated and triggered to secrete specific cytokines that attract inflammatory cells into the exposed epidermis, inducing rash, itching, and burning on the exposed skin surface [167].

Regarding in vivo testing, two test methods in guinea pigs—Guinea Pig Maximization Test (GPMT) of Magnusson and Kligman and Buehler test (OECD 406) [168]- and the Local Lymph Node Assay (LLNA—OECD 429) in mice [169] are available. Both induction and elicitation phases are addressed with the guinea pig. One difference between the guinea pig tests is the use of an adjuvant (Freund´s Complete Adjuvant) in the GPMT to potentiate skin sensitization; this is not done in the Buehler test. Considering the inability of microorganisms entering the intact skin due to their molecular size, the most important difference between the guinea pig tests is the intradermal injection in the GPMT *versus* the topical application in the Buehler test [168]. Furthermore, for some allergens, the Buehler test protocol was not sensitive enough to detect allergenicity [170]. Skin sensitization testing in mice is conducted via the LLNA by topical application of the test substance and the consideration of immunological events [169], which might be less sensitive for testing formulations containing microorganisms as active substance, for the same reason as the Buehler test. The LLNA considers the induction phase by inducing lymphocyte proliferation in the lymph nodes draining site of test substance application. This proliferation is proportional to the dose and to the potency of the applied allergen and provides a simple mean of obtaining a quantitative measurement of sensitization (OECD 429, [169]). If information on quantitative potency is required, the LLNA or appropriate non-animal methods—which are currently under discussion for their applicability—should be used to provide the necessary information on sensitization potency instead of the guinea pigs tests. Further advantages of the LLNA are the potential to reduce the number of animals and the capability of lowering pain and distress to the animals (OECD 429). In the LLNA the challenge-induced dermal hypersensitivity reactions and the use of an adjuvant are not required and therefore result in less pain and distress. Additionally, a reduced LLNA approach, which could use up to 40% fewer animals is also described as an option in this test guideline (OECD 429, [169]). Therefore, the LLNA has become the first choice within regulatory purposes for formulations with chemical active substances but has raised issues of false positive and false negative results [171]. These results may be explained by a lack of understanding of the mechanistic basis for the activation of compounds that are not directly protein-reactive, which may cause the various discrepancies between LLNA and GPMT [171]. Keeping these differences of the outcome in mind, the LLNA and GPMT can be carried out in a synergistic manner to evaluate skin sensitization [167]. If a test substance is defined as skin sensitizing by LLNA, there is no need to further validate this result by GPMT. The 70% accuracy of prediction of skin sensitization potential in human by conducting LLNA and GPMT and the fact that the outcomes of these tests are not always concordant [167] indicates the need for the development of further testing strategies. In particular it should be emphasized that both tests are in vivo methods making the development of alternative methods even more urgent. During the development of new predictive models, it is important not to be restrained by the understanding that a test by necessity must address all key events involved in the skin sensitization adverse outcome pathway. This point can be illustrated by the development of the LLNA, which was based on the understanding that sensitization involves clonal expansion of T-cells in the lymph node upon antigen presentation without the need to consider preceding events [172]. Currently, four key events in skin sensitization adverse outcome pathway are considered [173] and appropriate OECD-conform in vitro test methods have already been developed: key event 1 reflects the site of action and the covalent binding to skin proteins (OECD 442C) [174]; key event 2 reflects the generation of inflammatory cytokines and induction of cytoprotective genes by keratinocytes (OECD 442D) [175]; the third key event represents the activation of dendritic cells (OECD 442E) [176]. The final and fourth key event reflects the clonal expansion of T-cells in the site drained near where the test substance was applied (LLNA, OECD 429) [169].

The “Defined approaches on Skin Sensitization” describe methods (DPRA, KeratinoSens™, h-CLAT), for which transferability, within- and between-laboratory reproducibility have been characterized [177].

In respect of the validity of the in vivo tests, data from the recent renewal of the approval of the active substance CpGV in the EU leave room for discussion. Conducting the GPMT with two representative formulations, one formulation showed a positive result, probably due to the exceedance of the specific concentrations limits for two sensitizing impurities in the technical concentrate. For the second formulation a negative result was observed. The latter formulation did not include the potential sensitizers [178]. The validity of the above-mentioned in vitro tests for formulations and for formulations containing microorganisms as active substance have not been evaluated yet.

Beside the abovenamed in vivo and in vitro tests, there are gene expression-based test methods developed (e.g., SENS-IS assay, SenzaGen GARD®skin assay), purporting the determination of sensitizers and irritants, and which also may be able to assess potency. The GARD®skin assay was recently included in OECD 442E, for the evaluation of third key event, activation of dendritic cells [159].

Furthermore, there are in silico models to predict the skin sensitization potential of individual substances, but not that of formulations, and they are recently reviewed [167]. In a recent review article from Ta et al*.*, 2021, the various data sources are categorized by publications according to the collection of animal and non-animal data, as well as chemical structure information and the listing of online skin databases, which may be a useful tool to create predictive models [167]. Furthermore, commercial packages like a computer automated structure evaluation program, models based on animal tests, models based on non-animal tests, and a model based on mixed test types are described. Even the aspect of artificial intelligence is considered by reviewing machine learning-based models [167]. The appropriateness of these models for microorganisms as active substances has not been evaluated until now. However due to the size and composition of microbials, this could be challenging.

Existing skin sensitization assays evaluating the TGAI for microbial strains raise questions. Several issues on the applicability of microbials on existing skin sensitization alternative assays were presented and discussed during the OECD Conference on Innovating Microbial Pesticide Testing held in Paris, France in September 2022. The physical–chemical properties of microbials might be challenging, as the test material needs to be soluble and/or the quantity of the test material needs to be known. The assays require fluorescence or labeled detection methods which need to act with the microbial suspension. Standard procedure in validity testing is the use of controls. For microbials, no true positive or negative microbial controls exist as discussed earlier. Considering the adverse outcome pathway, a useful strategy needs to attempt to validate more than one study to test on the skin sensitizing potential, otherwise a true interpretation is not possible.

Therefore, two questions need to be answered considering the skin sensitizing potential of microorganisms: 1) Would validation of these NAMs be sufficient to mitigate the precautionary label statement? And 2) How do we address skin sensitization if the alternative methods are not able to answer the appropriate question to confirm study results.

##### Respiratory sensitization

Similar to microorganisms having the potential to be skin sensitizers, microorganisms also have the potential to provoke sensitization reactions by inhalation [179]. However it may not be the microorganism per se inducing the reaction but proteins, glycoproteins or secondary metabolites of the microorganisms [126]. For observed or reported sensitization reactions due to the use or microbial pesticides, the reporting on hypersensitivity incidents applies to OPPTS 885.3400 [180], which is related to the active substance.

An EFSA-sponsored literature review examined the respiratory sensitizing potential of microorganisms and their enzymes that are used as food/feed additives [181]. In this review article, current test methods were analyzed in respect of their validity. Considering in vivo inhalation animal models, the mouse is currently the best option. However, it suffers from several shortcomings that prevent it from being a reliable predictive model: most models require systemic sensitization by injection, but not inhalation, which is the most likely route of exposure for humans. In vitro models are currently not being used for predictive studies with biopesticides. Many cell lines are currently used in the laboratory; however, research is still trying to understand the role and mechanisms of the cells in the development of allergies, rather than using them to test the allergenicity of various molecules. The available in silico models can be useful to predict cross-reactivity between allergens. But they only take into account the structure of the allergen, which is only one of the many factors implicated in sensitization [181].

In the EU, an in vitro model for the prediction of respiratory sensitization of inhalable chemical active substances is currently in the submission process. The test method is an in vitro assay intended for the prediction of respiratory sensitization potential of airborne chemicals (including particulate matter and nano-materials). The method consists of a 3D in vitro system based on human cells cultured at the air–liquid interface (ALI). This system mimics the alveolar-capillary barrier and allows to assess substances with both low and high molecular weight (e.g. pollen or house dust mites) [182].

Furthermore, the EU-funded GARD®air project [183] has developed the first test for respiratory sensitization. The in vitro test should be capable of identifying chemical respiratory sensitizers. The test provides a binary prediction, classifying the test samples as either respiratory sensitizers or non-sensitizers and has a high specificity of 95% [184].

Lack of a validated assay to assess the respiratory allergy potential of biopesticides and pesticides with chemicals as active substance often requires manufacturers to include respiratory use in labeling. Some regulatory agencies require respiratory protective equipment, (for the US) typically a respirator with US National Institute of Occupational Safety and Health (NIOSH) prefix N-95, P-95, or R-95, for occupational handlers expected to have repeated exposures to the microbial pesticide. These measures are meant to lessen exposure that could lead to respiratory sensitization (US EPA, 2007 [185]), but the requirement on a microbial pesticide label can discourage its use, rather than encouraging it as an alternative to chemical pesticides. However, an assay evaluating biopesticides that could provide insight into a MPCA’s potential to induce respiratory allergy would be beneficial to both regulatory as well as biopesticide manufacturers by potentially allowing less restrictive labeling and a more accurate assessment of potential hazard. Thus, a study was designed with Bayer Crop Science and the US-EPA with the support of US EPA’s BPPD to investigate the Rat Basophil Leukemia cell (RBL) assay for its potential to identify microbial respiratory sensitizers. Using protocols established in the investigation of the allergic potential of molds [207,208] and the RBL assay as an index of antigen-specific, functional IgE (serum derived IgE antibodies) the potential allergenicity of two bacterial biopesticides was investigated. Previous studies with the RBL assay have demonstrated the potential to relative potency between various fungal microbes by providing an index of functional IgE induction by these microbes. Both biopesticides induced respiratory inflammation, but only multiple exposures induced IgE at statistically significant levels and only at doses calculated to be substantially higher than work related exposures. However, at this time the RBL assay does not meet the standards necessary to use it as a guideline regulatory study to determine if a respirator should be required, but does show promise as a tool for evaluating the potential for biopesticides to induce allergic responses. Further studies are needed to 1) identify commercially available allergy positive and negative controls; 2) evaluate reproducibility and transferability; 3) establish an acceptable cut-off for allergy induction.

Therefore, there is a need to develop a validated respiratory sensitization model to evaluate microbes to help identify if microbes are truly sensitizers and if respiratory protection should be used.

### Strategies used in various countries to assess skin and respiratory sensitization

In the EU, current regulations require a precautionary sentence on products containing microorganisms because of the lack of valid test methods, unless there is relevant information that there is no risk of sensitization or until a relevant test method has been validated with microbial products sensitization [86]. Authorizations granted in EU may therefore specify, as a non-specific risk mitigation measure, that PPE (e.g., masks) must be worn, taking into account the conditions of use, and that the exposure via inhalation to the plant protection product containing a micro-organism may be minimized. The US EPA requires a similar sentence: ‘Repeated exposure to high concentrations of microbial proteins can cause allergic sensitization’. Canada, as do other countries, has a similar approach, whereby it is standard for all microbial pesticides to be labelled as potential sensitizers.

A German proposal on labelling requirements for PPPs is presented here and is intended to initiate further discussions on this topic (see Table 3: German proposal for refinement of default MO sentence).

**Table 3:** German proposal for refinement of default MO sentence

Proposal	Proposed sentence	Explanation
for the label	“*May have the potential to cause skin and/or respiratory sensitization*”.	This phrase shall always be assigned as a precautionary statement (i.e., as a non-specific risk mitigation measure) to a PPP containing a microorganism; personal protective measures must be specified in these cases.
	“*May cause skin and/or respiratory sensitization*”.	This phrase shall be assigned to a PPP containing a micro-organism where there is clear evidence from experimental systems, documented human exposure or available scientific literature that the PPP may show sensitizing effects; if the attribution criteria is fulfilled for this second phrase, the first phrase will not apply. Personal protective measures must be specified in these cases.
for the active substance in the DAR/RAR	MAY HAVE THE POTENTIAL TO CAUSE SKIN AND/OR RESPIRATORY SENSITIZATION.	German colleagues are proposing to use in RAR/DAR templates for the active substance and the product (and **not on the label**) the following sentences to distinguish between the type of information on sensitization in the APPL/RMS assessment box of the DAR/RAR. The advantage is that the information from the study evaluation in the DAR/RAR is presented in a uniform and condensed way in the corresponding chapter.
	MAY CAUSE SKIN AND/OR RESPIRATORY SENSITIZATION and add the phrase “and there is evidence from animal studies (e.g., XY, 20XX) that the microorganism … is a skin /respiratory sensitizer”.	
	MAY CAUSE SKIN AND/OR RESPIRATORY SENSITIZATION and add the phrase “and there is evidence from human observations (e.g., XY, 20XX) that the microorganism … is a skin / respiratory sensitizer”.	
	MAY CAUSE SKIN AND/OR RESPIRATORY SENSITIZATION and add the phrase “and there is evidence from animal studies (e.g., XY, 20XX) and human observations (e.g., XY, 20XX) that the microorganism … is a skin / respiratory sensitizer”.	

Considering the German proposal, two options of PPE and for the resulting low-risk status may apply. First, PPE applies for the precautionary sentence according to Lichtenberg et al., 2015 [127] in DE. Since this kind of protection is non-specific, risk mitigation measures based on a precautionary sentence might not have an impact on the low-risk status of the microorganism. Second, in case there is clear evidence that the microorganism or components of it (proteins, glycoproteins or secondary metabolites) is a skin and/or respiratory sensitizer, specific risk mitigation measures are required and may be similar to that necessary for Skin Sens. 1 (H317; “May cause an allergic skin reaction”) and/or Resp. Sens. 1 (H334; “May cause allergy or asthma symptoms or breathing difficulties if inhaled”) according to Regulation (EC) No 1272/2008 [99]. This would then preclude the microorganism and the respective PPP from being considered of ‘low-risk’. Labelling with Skin Sens. 1 (H317) and Resp. Sens. 1 (H334) are not possible as the provisions of the classification and labelling framework for chemicals GHS cannot be used for microorganisms and thus they cannot be classified or labelled under this framework [179].

### Non-dietary exposure

Non-dietary exposure considers the exposure of operators, workers, bystanders and residents [186]. Operators might be exposed to biopesticides during the task of mixing and loading of the formulation and during application. Exposure of workers is estimated for activities that involve contact with treated crops. For seed treated products, the operator tasks of calibration, bagging and cleaning and the worker tasks of loading and sowing need to be assessed as well. Bystanders and residents are described as uninvolved third parties as they have no intentional contact with the biopesticide.

For the non-dietary exposure, the dermal route is the major exposure pathway, with inhalation route being minor exposure pathway, for operators, workers, bystander and residents. Four pathways of exposure are considered for bystander and resident risk assessment: spray drift, vapour, surface deposit and entry into treated crops [186]. Additionally, the exposure of a child and an adult will be assessed separately as bystanders.

The quantitative risk assessment for plant protection products containing chemical active substances is evaluated via the use of the reference values obtained from animal studies against non-dietary exposure to pesticides expressed in milligrams of the substance per kilogram body weight of the operator (covering also worker, resident and bystander; except for child assessment) [186]. Reference values are the Acceptable Operator Exposure Level (AOEL) considering sub-acute exposure and the Acute Acceptable Operator Exposure Level (AAOEL) describing acute exposure and take into account a single day. Exceedance of these reference values would require personal protective equipment and/or risk mitigation measures. Basis for non-dietary exposure assessment is the EFSA Guidance Document on the assessment of operators, workers, residents and bystanders [186]. This guidance describes the use of deterministic models when quantifying non-dietary exposure. The deterministic models were developed on the basis of field data. An online calculator reflects the guidance content. Within the alignment of reference values, the potential exposure without and with risk mitigation measures (e.g., PPE) can be estimated with the calculator.

Usually, no reference values were derived for microorganisms as no adverse effects will be observed in humans and/or in toxicological studies. If there are no adverse effects, then a non-dietary exposure assessment is not required. When less is known about metabolites or the microorganism, the Guidance on Risk Assessment of metabolites produced by microorganisms used as plant protection active substances proposes to a step-wise approach [101]. Step 1 considers the determination of the assessment type, Step 2 the collection of a basic set of information on metabolites, Step 3 the determination which of the identified metabolites are of concern and Step 4 the risk assessment for metabolites of concern.

The current practice for the risk assessment for metabolites of concern in the EU will be described using two examples. The secondary metabolite beauvericin is produced by *Beauveria bassiana* strain 203 and its genotoxic potential cannot be excluded based on in vitro and in vivo data. For the quantitative risk assessment, the use of Cramer Class III value of 0.0025 µg/kg body weight/day was discussed. However, EFSA re-considered the Threshold of Toxicological Concern (TTC) approach for beauvericin and concluded it is not applicable, since the TTC approach as proposed in the EFSA PPR Guidance on the Residue Definition for risk assessment has not been endorsed by risk managers and currently is not applicable for pesticides metabolites [90]. Finally, the use of PPE and respiratory protective equipment for operators and workers may be considered to reduce the non-dietary exposure (dermal and inhalation) due to the absence of a quantitative risk assessment [90]. The second example deals with *B. cereus*, which is known to cause food intoxications in humans. In 2016, the EFSA Panel on Biological Hazards (BIOHAZ) reviewed the risk for public health related to the presence of *B. cereus* and other *Bacillus* spp. including *B. thuringiensis* in foodstuffs [51]. The experts reviewed the classification and nomenclature of the *B. cereus* group, which is a subdivision of the *Bacillus* genus that consists of eight formally recognized species, inter alia,* B. thuringiensis.* Most cases of food-borne caused outbreaks have been associated with the *B. cereus* group and bacterial concentrations above 10^5^ colony forming unit (CFU)/g foodstuff. As *B. cereus* and *B. thuringiensis* strains are genetically closely related, these species are usually not discriminated between in routine clinical diagnostics or food microbiology. Following this, also some *B. thuringiensis* concentrations above 10^5^ CFU/g foodstuff may cause gastrointestinal diseases acc. to EFSA [51]. Operators, workers, bystander and residents may also come in contact via inhalation with *B. thuringiensis* after spray application of the formulation containing it. To estimate the non-dietary exposure, the German Federal Institute for Risk Assessment provided a proposal on a quantitative exposure assessment for microorganisms during the peer review of *B. thuringiensis* ssp. *aizawai* strain GC-91 (comment no. 4 on pages 463–464) [187].

The non-dietary exposure is assessed based on data provided under the Commissions Regulation 284/2013 / Commission Regulation (EU) 2022/1439 Section 7.5 [87, 88].

### Dietary exposure

Microbial plant protection products have the possibility, when applied on edible plants, to be consumed as viable microorganisms within its further chemical ingredients. They can multiply in the environment, on the food product and in the human and animal body. In addition, the metabolites produced by these organisms in each of these different environments can be consumed and have to be evaluated in the consumer risk assessment. For dietary exposure, the oral pathway needs to be considered to evaluate the consumer´s risk.

Reference values for dietary exposure for plant protection products containing chemical active substances are the Acceptable Daily Intake (ADI), which is an estimate of the amount of a substance in food or drinking water that can be consumed over a lifetime without presenting an appreciable risk to health [188]. The Acute Reference Dose (ARfD) is an estimate of the amount of a substance in food or drinking water that can be ingested over a short period of time, usually during one meal or one day, without appreciable health risk to the consumer [189]. Both ADI and ARfD are external reference values and are expressed on a body weight basis. Considering the dietary exposure, no risk mitigation measures are possible in case the reference values are exceeded.

A quantitative risk assessment will be conducted in case adverse effects are observed, otherwise this assessment is not required. Microorganisms can only be approved if they are not pathogenic, not infective under the recommended conditions of use, and not infective to humans according to Annex II of Commission Regulation (EC) No 1107/2009 and its amended version [82, 190]. If there is a concern for the microorganism related to its sensitizing potential, the precautionary sentence applies. And if there is a concern related to resistance – several treatment options with effective antimicrobials to protect human health are required [82, 190].

The assessment and evaluation of metabolites of concern follow the step-wise approach mentioned the section above according to the Guidance on Risk Assessment of metabolites produced by microorganisms used as plant protection active substances [101]. Two examples are used to describe the challenges for dietary exposure and dealing with microbial secondary metabolites. First, the compound 2,3-deepoxy-2,3-didehydro-rhizoxin (DDR) will be used. DDR is produced by the bacterial fungicide *Pseudomonas chlororaphis* strain MA 342 and may have genotoxic potential [191]. The potential genotoxic effect is still under discussion due to the insufficient data situation described in the conclusion by EFSA [191]. As a first-tier assessment, the TTC approach was used with an ADI of 0.0025 µg/kg body weight per day to estimate the dietary exposure. Basis for consumer risk estimation is the EFSA PRIMo – *Pesticide Residue Intake Model* [192]. This publicly available Excel-based calculation spreadsheet includes European food consumption data available for calculating dietary exposure. Within this model, the acute or chronic exposure of a compound reside will be aligned with a toxicological reference value. Considering the ADI of 0.0025 µg/kg body weight per day for DDR and the assumption of the DDR amount in cereals is 1 µg/kg body weight per day the TTC threshold will be exceeded by several orders of magnitude.

The second example considers swainsonine. This secondary metabolite will be produced by, among others, *Metarhizium brunneum*. Swainsonine was identified as a toxin in feed consumed by livestock. Animals consuming swainsonine have clinical observations pertaining to the nervous system due to the accumulation of glycoproteins, which symptomatically present as general incoordination, however the effects in the nervous system are permanent [193]. These findings were also confirmed in mice and rat studies [194]. Exposure to the toxicological relevant metabolite may either occur due to microbial pesticide application or due to its endophytic behavior: the ability to grow and colonize in plants. Swainsonine is water-soluble, rapidly absorbed, and expected to be widely distributed in the tissues of poisoned animals. The clearance from the tested tissues reveals half-lives of less than 20 h when containing 250 ng/g and 60 h when containing 2000 ng/g, respectively [195]. Considering the fast excretion of swainsonine, high daily dose levels might be necessary to cause chronic effects. Stegelmeier and co-workers, also determined dose response levels of sheep poisoned with locoweed containing swainsonine. The determined values could be used to derive an ADI for adults. According to their findings, the no observed adverse effect level (NOAEL) is 0.05 mg/kg body weight per day [196]. Considering this NOAEL and the inter-species factor of 10, as well as the intra-species factor of 10 and a factor of 3 to derive from a subchronic to a chronic exposure, an ADI value of 0.17 µg/kg body weight per day was derived. The obtained reference value is aligned with the exposure of the consumers. In EFSA´s conclusion a lack of qualitative and quantitative information for swainsonine was described [194]. In the dossier of *M. anisopliae,* the product analysis reveals no swainsonine concentration above the limit of detection (LOD) of 30 ppm (= 30 mg/kg) in the formulated product (comment no. 1 on page 383) [197]. While considering the application rate and the expected amount of swainsonine on the specific crop, the concentration of swainsonine may be calculated assuming the maximum concentration of 30 mg/kg swainsonine in the product. With this value the consumer risk can be estimated by using the EFSA PRIMo. This calculation does not include the potential in situ production. In the EU, the dietary exposure is assessed based on data provided under the Commissions Regulation (EU) 283/2013 / Commission Regulation (EU) 2022/1439 and 284/2013 / Commission Regulation (EU) 2022/1440 Sections 6 and 8, respectively [85–88].

## Challenges of the current guidelines and test methods

There are several challenges with the current testing guidelines for testing microbial pesticides. Because microbial pesticides not only possess unique properties that require special consideration in testing methods, the testing goals for understanding the hazard of microbial pesticides differ from those of chemical pesticides. Guidelines developed to assess hazards for chemical pesticides have limited applicability to substances that are comprised of living microorganisms. The primary difference is the need to assess infectivity and pathogenicity, in addition to toxicity of potential metabolites and sensitizing properties. While toxicity studies are well-designed and characterized for chemicals, microbial research is not often carried out to identify potential adverse effects and hazard. Therefore, the safety assessments conducted have been designed to evaluate potential adverse effects requiring exposure via routes relevant to cause pathogenicity (e.g., dietary, inhalation, contact exposure for fungi, etc.) as well as increased study duration to allow potential pathogenic effects, which are often delayed compared to toxic effects. Additionally, the exposure to microbial actives in the field may be difficult to estimate. Considering the initial external exposure (of the skin) during or shortly after application, it may be appropriate to use already developed standard models for exposure estimations. Considering the available standard models, the unit of the applied in-use solution in milliliter may be converted into CFU. It needs to be clarified, if toxicological values can be converted into CFU.

Testing can be organized in a tiered approach, where the lowest tier tests high concentrations of microbials, and testing does not advance to higher tiers unless adverse effects are observed. The following challenges have been noted while conducting the required guideline studies:

### Defining clearance of microorganisms

The term of “clearance” in these acute toxicity studies with the active substance may be a further point of discussion. It may raise these questions: 1) How is clearance defined? 2) Is it absolute elimination from the body or specific tissues or establishment of a distinct pattern of clearance? According to the regulations in the EU, there is no need to show 100% clearance to zero detected microorganisms or to show clearance measured in the tissues within a certain time frame. For spore-forming microbes, slow clearance is expected in the given observation period of the specific test guideline, but one must ask the question whether this increases hazard. Such a question does not necessarily trigger higher tier toxicity testing or diminish the chances for waiving other test requirements. The next issue to consider is how to evaluate the study, if there is no clearance defined, because the tested microorganism is an integral part of the intestinal flora (only of the tested animals, but not in human?). An increased focus may be put on the methodologies and level (what is enough? what percent of microbes are acceptable?) and time frame/speed (how long do we have to wait?) used to determine the amount of clearance. Currently, no harmonized test guideline exists to avoid such uncertainty. Furthermore, clearance may sometimes be not determined (equating with non-valid study in some regions?) and if determination occurs, it is challenging to interpret results of these studies due to the lack of a definition. Besides putting effort in the definition of clearance, the development of clearance evaluation standards would be helpful for evaluation. It may be discussed whether strong arguments for the exclusion of infectivity through the weight of evidence approach may apply and may be used for exclusion of clearance testing.

Another point of discussion may be the growth temperature in terms of whether growth temperature can be a parameter that can exclude infectivity and pathogenicity for humans and animals. In the report of the OECD/KEMI/EU Workshop on microbial pesticides: Assessment and management of risks in 2014, this issue was already addressed [198]. Microbial growth means multiplication in a tissue matrix: however, if organisms do not grow at certain temperature (so do not multiply), theoretically they could still remain hazardous (e.g., spores) as they could still be viable. Body temperature may also vary, as skin areas can have lower temperature than 37°C and microorganisms can adapt to a certain temperature range. So, selecting such a certain temperature has to be well considered. Recommendations were made, proposing at first that growth temperature cannot be an absolute parameter for not conducting infectivity studies and can only be used for human and warm blood vertebrates. Secondly, growth temperatures can be used to bridge (infectivity) data from one strain with data to another strain with limited data (read-across approach) in combination with other information (e.g., supported by phenotype similarity). Most recently, a threshold should be fixed for human temperature. Literature review, and in addition, a study on microorganism growth limit, should result in submission of a limited toxicological package (one infectivity study or no study at all) [198]. Therefore, a harmonized approach considering these recommendations would be helpful to overcome this issue including the application of this approach to a specific type of microorganisms, the setting of a temperature threshold in testing studies and to define the minimum data requirement for testing on human toxicity.

For future perspectives and keeping the 3-R principles in mind, it therefore can be proposed to use non-animal testing strategies to overcome the host-specificity and the question related to transferability to human and to reduce the tremendous challenges of result interpretation. A possible tool would be the data collection and data analysis via genome sequencing. With the help of whole genome analysis (WGS), the taxonomy of the specific microorganism may be assessed. WGS is not a data requirement, however, this tool is widely used by assessors for e.g., identity and taxonomy purposes and for the exclusion of specific genes related to metabolites or pathogenicity [101, 199, 200]. Further, genetic virulence factors described for the identified genus can be checked. This scientific information may be used to determine the relation to human pathogens in terms of infectivity and pathogenicity, and in lieu of conducting in vivo studies.

### Data interpretation

The US EPA’s Series 885 guidelines were written to be flexible and applicable to a wide range of microorganism test substances. However, this flexibility provides little guidance on the execution and interpretation of these studies. Guidance could be useful to consider the use of controls in the studies.

For example, the results of a pathogenicity study are not defined as to what is considered “positive”. While it may be clear that a “positive” finding in a pathogenicity study demonstrates signs of disease or infection in the tissues, the studies are not required to determine the cause, just requiring moving to a higher tier study. Results may be inaccurate or difficult to interpret, delay the registration process due to the need for additional testing, or result in failure to sufficiently support the safety of these pesticidal substances and their products for registration. For the purpose of study interpretation, including or not including a positive control in a pathogenicity study also raises some challenges. Positive control microbes have not been validated in the test system and could have a very different mode of action to causing disease compared to the microbe or fungus, therefore, a positive control does not necessarily aiding in the study interpretation. The use of a chemical positive control might offer some adverse effects: however, the test materials are vastly different and does not help to differentiate effects due to particles or cellular debris.

Furthermore, the lack of a negative control is challenging, as often to control animals saline or carboxymethyl cellulose is administered as a surrogate for the whole broth. It is not uncommon to see some effects in the test group animals, especially in the intratracheal pathogenicity studies. Therefore, it must be determined if the effects are caused by the microorganism or if they are unspecific effects caused by the administration of an inert (biological) material (e.g., autoclaved spores) directly to the lungs. The need to consider the nature of the test material is critical– sometimes effects are observed, but if negative control is not the same form (e.g., negative control is a liquid-based carrier and contains no cells) then it can be difficult to draw any useful scientific conclusions.

In respect of testing on infectivity, inactive autoclaved or radiation of test material may be fit as negative control. The observation of no effects in the group tested with the inactive autoclaved material compared to no observed effects in the group tested with the active microorganism may be a hint for no evidence for infectivity excluding effects after in situ replication.

There can be challenges in meeting some of the Food and Agriculture Organization of the United Nations (FAO) requirements for specifications of physical properties due to the complex mixture that microbial pesticides consist of. There has been adoption by FAO in the forthcoming release of a dedicated manual for the development and use of microbial pesticide specifications but still there are some authorities that are not applying the flexibility. In general, greater tolerances are needed for specifications due to the fermentation production of living microbial product.

### Dose concentration/limit dose

The CFU concentrations to be employed in the test vary among MPCAs, depending upon the nature of the test material. However, if the CFU concentrations are increased in the test compared to the concentration in the formulation, it could result in observed adverse effects. These effects may be misinterpreted as pathogenic effects as there were associated with the amount of test material administered to the animal rather than due to the microorganism itself. Moreover, the increase of CFU concentration in the test may be a major challenge to meet the OPPTS 885 series guidelines (10^8^ CFU for oral and intratracheal studies, and 10^7^ CFU for i.p./i.v. studies). If one needs to reduce the CFU concentration, the dose may not be considered meeting the study requirements. Therefore, the reduction of CFU concentration in the test compared to the formulation and to meet the guidelines has to be explained in detail for justification. If the dose concentration is reduced to avoid negative effects or death in the test animals, one should ask if this leads to comparable results in the hazard assessment of different microorganism. While opportunities for alternative limit CFU dose justifications are allowed, technical interpretations for the physical properties of all microorganisms are not readily available.

### Secondary metabolites

Microorganisms are known to produce primary and secondary metabolites [201]. Primary metabolites are directly involved in general metabolism required for basic life procedures such as growth, development and reproduction of a microorganism and are typically key components in maintaining normal physiological processes. Primary metabolites are generally not metabolites of potential concern [101]. Secondary metabolites are not essential for the primary metabolic processes of microorganisms and show numerous biological activities possibly related to survival functions of the microorganism, such as competition, parasitism or symbiosis and metal transport [101]. Secondary metabolites are produced by the microorganism under specific physical and biological conditions.

Secondary metabolites can be produced either before application (i.e., in the fermentation broth, during the manufacturing of the active substance) or in situ after application (i.e., in the field or in the greenhouse). The secondary metabolites produced in situ may be taken up by the plant or may be present on parts of plants or even produced inside the plant due to endophytic growth properties of the strain. Information about the persistence of the secondary metabolite and toxicological reference values should be used to assess the relevance of the in situ production for evaluating the operators/workers/bystanders/residential exposure risk.

During microorganism evaluation data on produced secondary metabolites will be collected. Then, data of secondary metabolites that may pose a risk to human health will be identified. The potential risk will be evaluated by using the respective data requirements and the Uniform Principles for microorganisms [85, 202]. The production of metabolites of concern is based on the capacity of the individual strain to express such metabolites [101]. To minimize the toxicity testing series for secondary metabolites, their identification in the active substance may be waived by evaluation of the genome (WGS). When secondary metabolites are produced by the microorganism during fermentation, they are arguably part of the TGAI, and the toxicity tests conducted with the formulation have evaluated acute toxicity of the metabolites; thus, it can be argued that no further testing with the secondary metabolites on acute toxicity is needed if toxicity tests with the TGAI or whole broth are available.

Production of secondary metabolites in situ may be difficult to investigate and very limited literature precedence exists for such investigations for unknown metabolites. For known metabolites analytical methods are available which allow monitoring in the field. In the EU, a new guidance document is available which provides a practical approach on how the data requirements on metabolites can be applied in the approval of microorganisms as active substances at EU-level and the authorization of plant protection products at Member State level [101]. This guidance [101] addresses metabolites present in the active substance and the plant protection product and also those produced by the microorganism after application. The OECD working document (Series on Pesticides No. 98 [201, 203]) provides testing strategies for secondary metabolites present in the growth media and draws the attention to the fact that a tiered system involving acute, mesocosm and field testing may be required for microorganisms as well as information on the already existing natural background levels.

The importance of secondary metabolites was outlined in a workshop on microbial pesticides in 2014 [198]. It was stated that there is a lack of guidelines to address the data requirements with regard to metabolites. In view of the former data requirements several of the EFSA conclusions raise concerns of issues that could not be finalized due to the potential production of toxins/secondary metabolites of (often) unknown toxicity. WGS bioinformatic analysis could be of help to identify the possibility of production of hazardous secondary metabolites. Their actual production can be investigated in function of the growth cycle of the microorganisms (logarithmic/stationary phase) and can be detected and quantified by chemical analytical tools.

As an example, a prominent secondary metabolite is beauvericin, which is produced by *Beauveria bassiana* strain 203 [90]. Beauvericin is a toxin produced and stored intracellulary and which is not secreted. However, the genotoxic potential of beauvericin cannot be excluded based on in vitro and in vivo testing [90]. If secondary metabolites are identified as genotoxic, then management options are the used tool in risk assessment by e.g., the restriction of the intended uses. Considering beauvericin, the intended use in ornamental palm trees was accepted only and “the level of the metabolite beauvericin in the formulated product […] shall not exceed 80 μg/kg” [204].

### Good laboratory practice status

Studies to be submitted for registration need to be conducted under Good Laboratory Practice (GLP) when they are related to human health in the EU and US, preferably with GLP test materials. In some instances, early development studies, which may be used for regulatory submissions, are conducted under non-GLP. In addition, there can be instances where generating accurate concentration and homogeneity of the test material is difficult, due to the physical properties of the test material. Therefore, expert judgement is needed for the use of such non-GLP studies in evaluation and risk assessment. In the EU, the provisions on GLP stated in Commission Regulation (EU) No 2022/1439 amending Commission Regulation (EU) No 283/2013 shall be applied [85, 86].

## Conclusion and outlook

In summary, as the increase in interest in alternatives for synthetic pesticides grows, the test methods that were developed for assessing hazards for biopesticides years ago need revisiting to ensure they are still fit for purpose for human health assessments. Today, the application of NAMs in regulatory risk assessment continues to be an important area of development. Therefore, the authors suggest to consider developing NAM strategies for microbials where applicable. For chemical regulation purposes two very interesting papers about NAMs were published in the last year. One paper describes the research, development and validation activities, as well as initiatives that promote the uptake and use of non-animal methods and approaches in science and regulation in the EU [205]. The other lists several NAMs considered by US EPA under the Toxic Substances Control Act [206]. Both papers may be relevant for consideration and adaptation for microbial regulation, however, due to the biological nature of microbials, it is not as simple as using the validated NAMs for synthetic pesticides to directly transfer to evaluate microbials. In addition, as technological advances have evolved that are specific to microbial products (ie availability of WGS), these strategies should be incorporated to identify and address potential hazards without the use of animal models. Furthermore, harmonization of testing and evaluation strategies is also critical for the success of global microbial product registrations.

## Data Availability

No datasets were generated or analysed during the current study.

## Abbreviations

ADI: Acceptable Daily Intake
ALI: Air–liquid interface
AOEL: Acceptable Operator Exposure Level
AAOEL: Acute Acceptable Operator Exposure Level
ARfD: Acute Reference Dose
ATE: Acute toxicity estimate
BIOHAZ: EFSA Panel on Biological Hazards
BPPD: Biopesticides and Pollution Prevention Division
CAGR: Compound annual growth rate
CFU: Colony Forming Unit
CLP: Classification, Labelling and Packaging
CpGV: Cydia pomonella granulovirus
DDR: 2,3-Deepoxy-2,3-didehydro-rhizoxin
EFSA: European Food Safety Authority
EU: European Union
FAO: Food and Agriculture Organization of the United Nations
GHS: Globally Harmonized System of Classification and Labeling of Chemicals
GLP: Good Laboratory Practice
GPMT: Guinea Pig Maximization Test
IATA: Integrated Approach to Testing and Assessment
ICCVAM: Interagency Coordinating Committee on the Validation of Alternative Methods
KEMI: Swedish Chemicals Agency (Kemikalieninspektionen)
LLNA: Local Lymph Node Assay
LOD: Limit of Detection
MPCAs: Microbial Pest Control Agents
NAMs: New Approach Methods
NIOSH: US National Institute of Occupational Safety and Health
NOAEL: No Observed Adverse Effect Level
NPV: Nucleopolyhedroviruses
NRU: Neutral Red Uptake
OECD: Organization for Economic Cooperation and Development
OPPTS: Office of Prevention, Pesticides, and Toxic Substances
PPE: Personal protective equipment
PPP: Plant Protection Products
PRIMo: Pesticide Residue Intake Model
REACH: Registration, Evaluation, Authorisation and Restriction of Chemicals
RhCE: Reconstructed Human Cornea-like Epithelium
RMS: Rapporteur Member State
TGAI: Technical grade active ingredients
TTC: Threshold of Toxicological Concern
US: United States
US EPA: United States Environmental Protection Agency
WGS: Whole Genome Sequencing
